# FUT6 Suppresses the Proliferation, Migration, Invasion, and Epithelial–Mesenchymal Transition of Esophageal Carcinoma Cells via the Epidermal Growth Factor Receptor/Extracellular Signal-Regulated Kinase Signaling Pathway

**DOI:** 10.5152/tjg.2024.23604

**Published:** 2024-09-01

**Authors:** Jianle Lao, Yanmin Pang, Hongming Chen, Xiqiang Tang, Rizhu Li, Danlei Tong, Ping Qiu, Qianli Tang

**Affiliations:** 1 Department of Surgery, Jinan University, Guangzhou, Guangdong Province, China; 2 Department of Cardiothoracic Surgery, The Affiliated Hospital of Youjiang Medical University for Nationalities, Baise, Guangxi Province, China; 3 Key Laboratory of Tumor Molecular Pathology of Baise, The Affiliated Hospital of Youjiang Medical University for Nationalities, Baise, Guangxi Province, China; 4 Department of Hematology, The Affiliated Hospital of Youjiang Medical University for Nationalities, Baise, Guangxi Province, China; 5 Department of Surgery, Graduate School of Guangxi University of Traditional Chinese Medicine, Nanning, Guangxi Province, China; 6 Life Science and Clinical Research Center, The Affiliated Hospital of Youjiang Medical University for Nationalities, Baise, Guangxi Province, China

**Keywords:** Esophageal cancer, *FUT6*, EGFR/ERK signaling pathway, epithelial-to-mesenchymal transition

## Abstract

**Background/Aims:**

Esophageal cancer (ESCA) is a high-incidence disease worldwide, of which the 5-year survival rate remains dismal since the cellular basis of ESCA remains largely unclear. Herein, we attempted to examine the manifestation of fucosyltransferase-6 (*FUT6*) in ESCA and the associated mechanisms.

**Materials and Methods:**

The GSE161533 dataset was used to analyze a crucial gene in ESCA. The expression of *FUT6* was investigated in normal esophageal epithelial cells and ESCA cell lines. Following *FUT6* knockdown or overexpression, cell proliferation, migration, invasion, and levels of epithelial–mesenchymal transition (EMT)-related and epidermal growth factor receptor (EGFR)/extracellular signal-regulated kinase (ERK) signaling pathway-related proteins were evaluated using CCK-8, Transwell, and Western blotting with antibodies against EGFR, p-EGFR, E-cadherin, Vimentin, N-cadherin, ERK1/2, and p-ERK1/2), respectively. EGF was administered to stimulate the EGFR/ERK signaling pathway, followed by the assessment of cellular activity.

**Results:**

Database analysis revealed that *FUT6* was downregulated in the ESCA cells. Our study indicated that *FUT6* is suppressed in various ESCA cell lines. Moreover, cell proliferation, invasion, migration, and EMT-related protein levels were conspicuously enhanced or restrained by *FUT6* disruption or overexpression. *FUT6* overexpression suppressed the malignant activities of the cells when stimulated by EGF, including inhibition of cell growth, movement, invasion, and EMT advancement, as well the reduction the levels of EGFR/ERK pathway proteins.

**Conclusion:**

In conclusion, *FUT6* can suppress the EGFR/ERK signaling pathway activated by EGF, leading to the potential attenuation of ESCA cell proliferation, invasion, migration, and EMT.

**Main Points**

- Fucosyltransferase-6 (FUT6) was identified as a key gene in esophageal cancer (ESCA).
- FUT6 was downregulated in ESCA.
- FUT6 contributes to preventing ESCA cell proliferation, migration, and invasion.
- FUT6 inhibits epithelial–mesenchymal transition (EMT) and the epidermal growth factor receptor (EGFR)/extracellular signal-regulated kinase (ERK) signaling pathway in ESCA cell lines.
- FUT6 suppressed the cell viability and EMT of ESCA cell lines via the EGFR/ERK signaling pathway.

## Introduction

The 2020 Global Cancer Statistics report revealed that esophageal cancer (ESCA) has emerged as a worldwide malignant neoplasm, with a staggering 6 604 100 new cases, constituting 3.1% of all newly diagnosed cancers.^1^ Esophageal cancer is a high-incidence disease in China, with 150 000 deaths per capita in the whole year, ranking fourth in the prevalence of malignant tumors.^2^ Due to the lack of symptoms and typical clinical manifestations in the early stage of the disease, most patients have been diagnosed at advanced stages at the time of diagnosis, often leading to poor prognosis. Consequently, the overall survival rate for patients with ESCA after 5 years is 20%.^3^ The development of ESCA is highly intricate, and as advancements in molecular biology continue, our understanding of its pathogenesis is consistently developing.^4^ However, the successful clinical application of these molecules to improve the management of patients with ESCA is still far away. Therefore, further elucidation of ESCA pathogenesis and identification of other more effective molecular markers are particularly important for the occurrence and development of ESCA and may be very helpful for screening other high-risk groups.

The epithelial-to-mesenchymal transition (EMT) is a crucial process involved in the advancement and spread of tumors.^5^ Epithelial-to-mesenchymal transition is a constantly changing process where epithelial cells transform, losing their original characteristics, and adopting a mesenchymal phenotype. This change occurs through the elimination of E-cadherin or cytokeratin (epithelial markers) and the acquisition of N-cadherin or vimentin (mesenchymal markers).^6^ These alterations involve changes in morphology, including restructuring of the cytoskeleton, interference with the cell’s ability to bind to other cells and the matrix, and the absence of cell polarity.^7^ Collectively, these occurrences enhance tumor cell invasion, migratory properties, and ultimately metastasis. Currently, sufficient evidence demonstrates the key role of EMT during ESCA development and progression. Therefore, this study attempted to screen out potential indicators for the management, including diagnosis and treatment of ESCA, by using EMT as an entry point.

DNA microarrays are now widely used as powerful tools for detecting cancer, enabling rapid identification and classification of cancers, and detecting cancer-related genes for early diagnosis and treatment.^8^ Currently, DNA microarray technology is an important means for high-throughput screening of differentially expressed genes and has been widely used in many fields, including scientific research, drug screening, and clinical diagnosis. Accordingly, in this study, GSE161533 was obtained after searching the ESCA-related microarray dataset using the GEO database. After analyzing the microarray samples, it was found that fucosyltransferase 6 (*FUT6*) was downregulated in tumor tissue samples of patients with ESCA, suggesting that *FUT6* may be a key gene affecting the behavior of cells in ESCA. A previous study indicated that *FUT6* functions as a gene that inhibits the development of head and neck squamous cell carcinoma as well as breast cancer.^9,10^ Nevertheless, information regarding the involvement of *FUT6* in ESCA is lacking.

In this study, the combination of the GEPIA online database and the GSE161533 microarray dataset was applied to investigate the *FUT6* expression profile in tumor tissue samples of patients with ESCA. Subsequently, the effects of *FUT6* on the proliferation, migration, invasion, and EMT of ESCA cells were explored based on ESCA cell lines with *FUT6* knockdown and overexpression. This study aimed to provide a new target for finding diagnostic indicators and developing drugs for the management of ESCA.

## Materials and Methods

### Bioinformatics Analysis

The ESCA-related microarray datasets were chosen from the GEO database, the world’s largest and most extensive public gene expression database, accessible at https://www.ncbi.nlm.nih.gov/geo/. The microarray matrix file GSE161533 contains 84 samples of normal and para-tumor and tumor tissue samples from 28 patients with ESCA, downloaded from the GPL570 platform. The differential expression analysis was processed based on the normal and tumor tissue samples through the analysis tool in GEO, GEO2R, with the threshold set as FDR < 0.05 and |Fold Change| ≥ 1.3.

The expression levels of *FUT6* were also investigated based on the GEPIA database (http://gepia.cancer-pku.cn/), which contains 182 ESCA tumor tissue samples and 286 paired normal tissue samples.

### Cell Culture

Human ESCA cell lines (KYSE-30, OE-19, KYSE-150, and TE-1) and Het-1A, a normal esophageal epithelial cell, were purchased from Procell (Heidelberg, Germany). Cells were maintained in Dulbecco’s modified Eagle medium (DMEM) supplemented with 10% fetal bovine serum (Gibco, USA) and 1% (v/v) penicillin–streptavidin (cat. no. P4333, Sigma-Aldrich, USA) and cultured at 37°C under 5% CO_2_. To achieve the activation of the EGFR/ERK signaling pathway, EGF (cat. no. SRP3027, Sigma-Aldrich; final concentration, 50 ng/mL) was added to the medium for 24 hours of incubation, while 1% bovine serum albumin (BSA; cat. no. 9048-46-8, Sigma-Aldrich) was used as the negative control for EGF.

### Cell Transfection

The shRNA designed using the full-length *FUT6* gene sequence (Gene ID: 2528) and the pcDNA-FUT6 recombinant plasmid constructed using purified *FUT6* cDNA were designed by Shanghai Jima Biotechnology Co., Ltd. (Shanghai, China). Briefly, when the cells grew to 50%-75% confluency, overexpressed *FUT6* vector (oe-FUT6) and sh-FUT6 were transfected into TE-1 and OE-19 cells using Lipofectamine 3000, respectively. In each well, 50 nmol/L shRNA or pcDNA and 2 μL liposomes were dissolved in 100 μL serum-free DMEM medium, respectively, and the above 2 liquids were gently mixed for 5 minutes and then incubated for 20 minutes. Next, 1 mL of DMEM medium combined with the above mixture was added to each well for overnight incubation at 37°C. The untransfected group (control) and the transfected empty vector interference groups (oe-NC and sh-NC) were set up for the following experiments 72 hours post transfection.

### Cell Proliferation Assay

TE-1 and OE-19 cells were seeded into a 96-well plate at a cell density of 2 × 10^3^ cells/well. Following incubation for 24, 48, and 72 hours, 10% (v/v) of CCK-8 solution (cat. no. CK04, Dotsujima, Japan) was introduced for a further 2-hour incubation at 37°C. Subsequently, the absorbance at 450 nm was assessed with a microplate reader.

### Cell Migration and Invasion Assays

Transwell chambers (cat. no. CLS3470, Corning, USA) were positioned on a 24-well plate to conduct the cell migration and invasion assays. Subsequently, the upper chamber was seeded with cells and added to serum-free DMEM, whereas the lower chamber was supplemented with DMEM containing 10% (v/v) fetal bovine serum. After 24 hours, the cells were subjected to staining using a solution of 0.1% crystal violet. The upper chamber was cleared of unmigrated cells using a cotton swab after treatment with 5% crystal violet solution made with methanol. Using a microscope, photographs of 3 separate areas were taken, and cell counts were conducted. For the cell invasion assay, the steps are similar to the migration assay except the Transwell chamber was pre-coated with 50 µL of diluted Matrigel.

### Reverse Transcription-Quantitative Polymerase Chain Reaction (RT-qPCR)

After extracting total RNA from cells with Trizol reagent, RNA was converted into cDNA with a reverse transcription kit (cat. no. A3500; Promega, USA) according to the manufacturer’s instructions, followed by carrying out experiments with SYBR PremixEx Taq kit (cat. no. RR420A, TaKaRa, Japan). Based on the 2^−ΔΔCT^ method, the relative expression of *FUT6* mRNA was calculated, with *GAPDH* serving as an internal reference. The RT-qPCR primers were as follows: *FUT6* (Forward—ATGTGGCCCCTGGGTTTATG, Reverse—CTGTTTGGTTCTGCAACGGG) and *GAPDH* (Forward—TGTAGGCTCATTTGCAGGGG, Reverse—TCCCATTCCCCAGCTCTCAT).

### Western Blotting

The gathered cells were rinsed thrice using phosphate-buffered saline and then broken down using protease and phosphatase inhibitors in radioimmunoprecipitation assay lysate (Cell Signaling Technology, USA) for 10 minutes while kept on ice. The Bio-Rad protein assay supplied by Hercules was applied to determine protein concentrations. An equal quantity of protein samples was separated using 12% SDS-PAGE. Subsequently, the samples were transferred to polyvinylidene difluoride membranes (Millipore, USA) prior to 1 hour blocking with 5% skim milk. Primary antibodies against *FUT6* (cat. no. abs106810; Absin, USA), E-cadherin (cat. no. AF0138; Beyotime, China), N-cadherin (cat. no. AF0243; Beyotime), Vimentin (cat. no. AF0318; Beyotime), EGFR (cat. no. AF5153; Beyotime), ERK1/2 (cat. no. AF1051; Beyotime), P-EGFR (cat. no. AF5794; Beyotime), P-ERK1/2 (cat. no. AG2954; Beyotime), and GAPDH (cat. no. AF0006; Beyotime) were incubated overnight at 4°C. Afterward, the secondary antibodies (1:3000, Santa Cruz) were incubated for 1 hour at ambient temperature. Following electrochemiluminescence imaging, grayscale analysis was conducted using Image J software, while Image-Pro Plus was used to examine optical density. The protein expression levels in each group were determined by comparing them with those of GAPDH, which served as an internal reference. The relative content of the target protein in the control group was set to 1. The experiment was repeated thrice.

### Statistical Analysis

Statistical analysis was performed using GraphPad Prism version 8.0. (GraphPad Software, Boston, Massachusetts USA) and the data in this study are presented as the mean ± SEM. To assess the significant disparity between the 2 groups, the *t*-test was employed. Differences between more than 2 groups were analyzed using ANOVA. A significance level of less than .05 (*P* < .05) was considered statistically significant.

## Results

### *FUT6* Was Downregulated in ESCA

**Figure 1.**
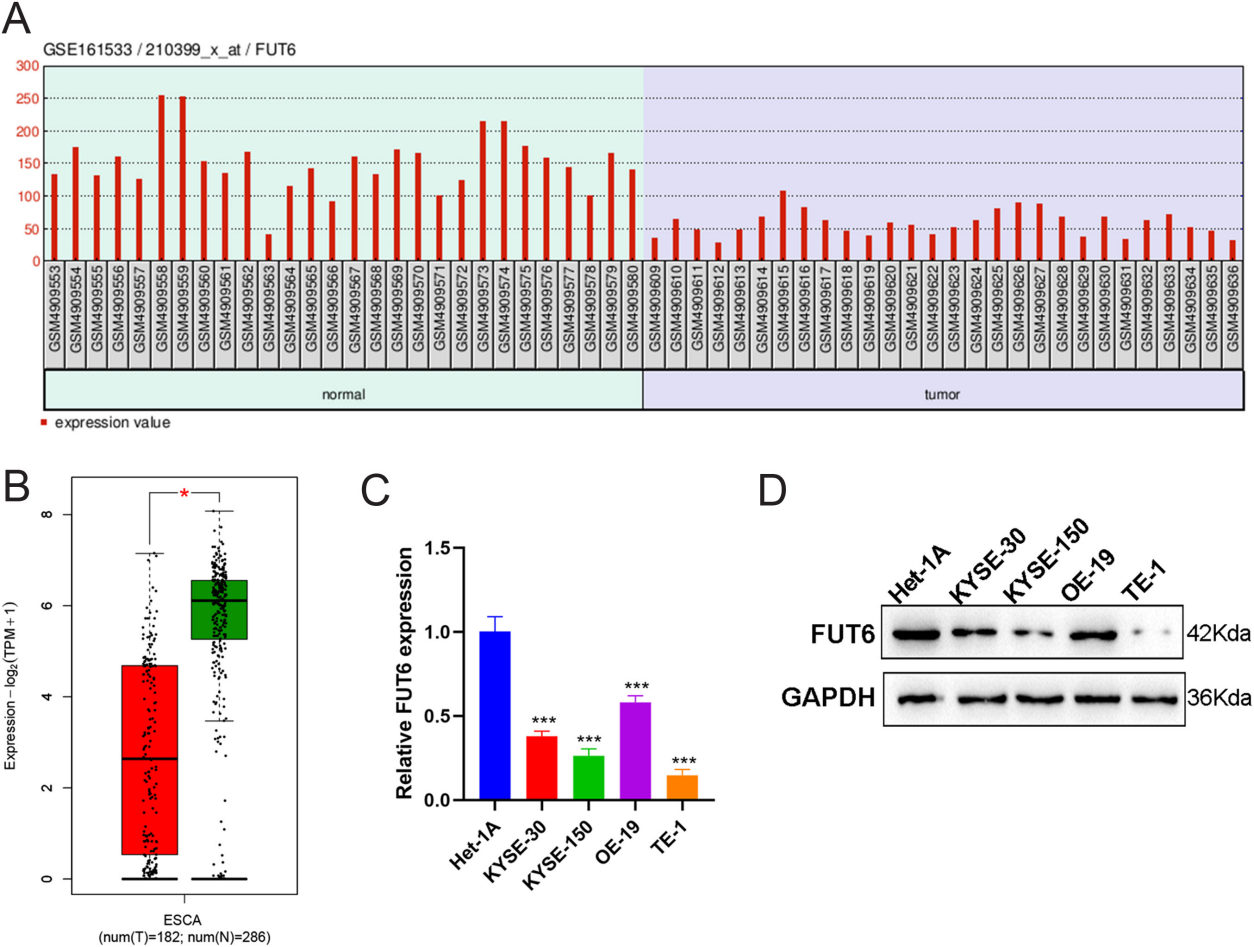
*FUT6* was downregulated in ESCA. (A) Based on the GSE161533 microarray dataset, the *FUT6* expression in 28 paired normal tissues and tumor tissues from 28 patients with ESCA was analyzed using GEO2R. (B) Box plots of differential expression of *FUT6* in 182 ESCA tumor tissues and 286 normal tissues were obtained based on the GEPIA database. (C) RT-qPCR measurement of the *FUT6* mRNA levels in KYSE-30, KYSE-150, OE-19, TE-1, and Het-1A cell lines. (D) Western blotting of the *FUT6* protein levels in KYSE-30, KYSE-150, OE-19, TE-1, and Het-1A cell lines. Data: mean ± SEM. N = 3. ***P < .001 vs. the Het-1A group.

In this study, we obtained the ESCA-related microarray dataset GSE161533 from the GEO database and found that *FUT6* expression was downregulated in tumor tissue samples from patients with ESCA using GEO2R analysis (Figure 1A). Similarly, the examination using the GEPIA data indicated a significant decrease in *FUT6* expression in the 182 ESCA tumor samples compared with the 286 corresponding normal or para-cancerous tissues (Figure 1B). To confirm this discovery, four distinct ESCA cell lines (KYSE-150, KYSE-30, OE-19, and TE-1) and esophageal normal epithelial cells (Het-1A) were cultivated. Subsequently, *FUT6* expression levels in each cell line were assessed using RT-qPCR and Western blotting. As shown in Figures 1C and 1D, *FUT6* expression was significantly reduced in all ESCA cell lines compared with Het-1A. This observation aligns with the findings obtained from the analysis of GSE161533 microarray and GEPIA data.

### The Role of *FUT6* in the Proliferation, Migration, and Invasion of ESCA Cell Lines

**Figure 2.**
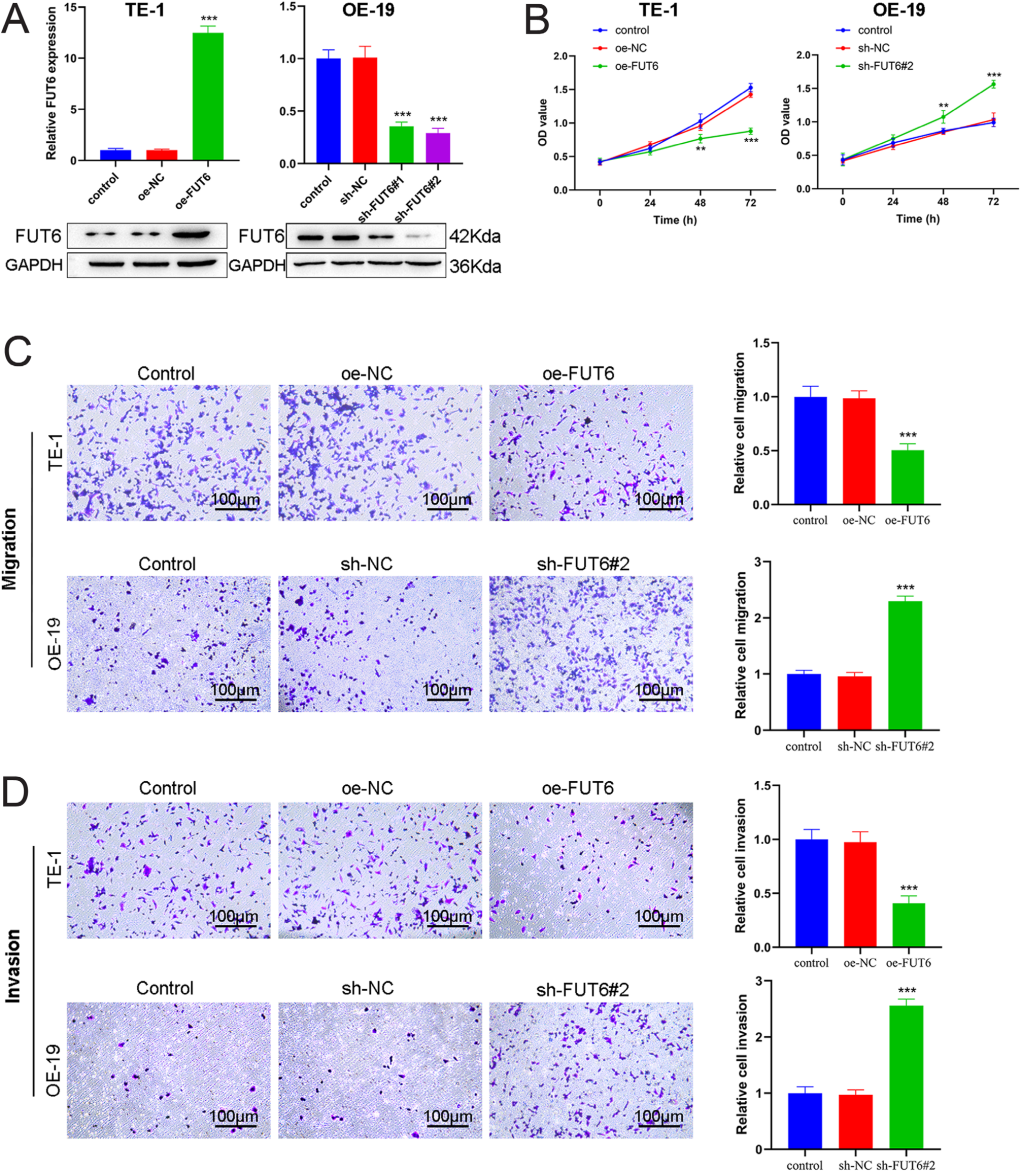
*FUT6* contributes to the prevention of the proliferation, migration, and invasion of ESCA cells. (A) RT-qPCR and Western blotting of the *FUT6* mRNA and protein levels in TE-1 or OE-19 cells, transfected with oe-FUT6 or sh-FUT6#1 and sh-FUT6#2. (B) CCK-8 assay check of the proliferation ability of TE-1 and OE-19 cells, transfected with oe-FUT6 or sh-FUT6#2, respectively. (C) Transwell assay check of the migration ability of TE-1 and OE-19 cells, transfected with oe-FUT6 or sh-FUT6#2, respectively. (D) Transwell assay check of the invasion ability of TE-1 and OE-19 cells, transfected with oe-FUT6 or sh-FUT6#2, respectively. Data: mean ± SEM. N = 3. **P < .01, ***P < .001 vs. oe-NC or sh-NC group.

To explore whether *FUT6* contributes to the malignant behaviors of ESCA cells, based on *FUT6* expression in ESCA cell lines, *FUT6* was knocked down by transfection with shRNA in the OE-19 cell line, which had the highest expression of *FUT6*, while the TE-1 cell line, with the lowest expression of *FUT6, *was overexpressed *FUT6.* Figure 2A shows that *FUT6* expression levels were increased 12-fold after transfection with the oe-FUT6 overexpression vector in the TE-1 cell line. Two different shRNAs were selected to target *FUT6* knockdown to avoid off-target effects, and sh-FUT6#2 displayed a higher knockdown efficiency than sh-FUT6#1; therefore, sh-FUT6#2 was applied for the follow-up study (Figure 2A). In the clarification of the role of *FUT6* in ESCA, CCK-8 and Transwell assays revealed that the cell proliferative (Figure 2B), migration (Figure 2C), as well as invasive (Figure 2D) abilities were all reduced after stable overexpression of *FUT6* in the TE-1 cell line. However, the optical density value and the number of cells crossing the Transwell chamber were significantly increased after *FUT6* knockdown in the OE-19 cell line (Figure 2B-D). These findings indicate that *FUT6* may contribute to the prevention of ESCA growth and metastasis.

### *FUT6* Is Involved in EMT and the EGFR/ERK Signaling Pathway in ESCA Cell Lines

**Figure 3.**
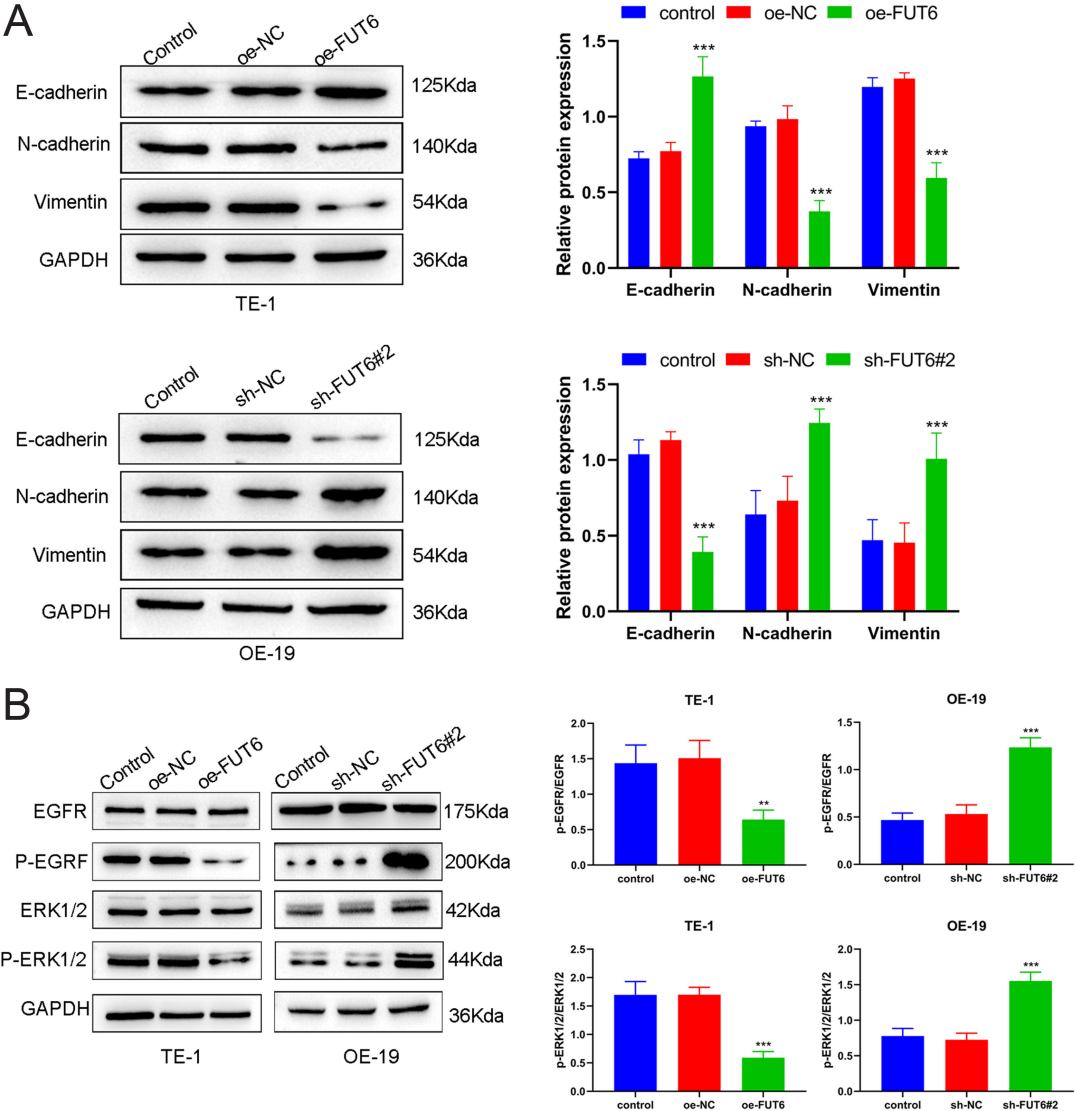
*FUT6* inhibits EMT and the activation of the EGFR/ERK signaling pathway in ESCA cells. (A) Western blotting of the expression levels of E-cadherin, N-cadherin, Vimentin proteins in TE-1 and OE-19 cells, transfected with oe-FUT6 or sh-FUT6#2, respectively. (B) Western blotting of the protein levels of p-EGFR, EGFR, p-ERK1/2, and ERK1/2 proteins in TE-1 and OE-19 cells, transfected with oe-FUT6 or sh-FUT6#2, respectively. Data: mean ± SEM. N = 3. **P < .01, ***P < .001 vs. oe-NC or sh-NC group.

Epithelial-to-mesenchymal transition is widely recognized as a crucial process that drives tumor advancement, including metastasis. In addition, the signaling of epidermal growth factor receptor (EGFR) significantly affects EMT in various cancers, including ESCA. Therefore, this study delved deeper into the impact of abnormal *FUT6* expression on proteins associated with EMT and the EGFR/ERK signaling pathway in ESCA cell lines. In the TE-1 cell line, Western blotting results revealed that *FUT6* upregulation significantly enhanced the levels of E-cadherin protein while reducing the levels of N-cadherin and Vimentin proteins, whereas the above proteins were expressed in the opposite pattern in the OE-19 cell line with *FUT6* knockdown (Figure 3A). In addition, *FUT6* overexpression preeminently contributes to suppressing the EGFR/ERK signaling pathway, as evidenced by the decreased protein levels of p-EGFR and p-ERK1/2. In contrast, significantly increased p-EGFR and p-ERK1/2 protein levels were observed after *FUT6* knockdown (Figure 3B). Altogether, *FUT6* inhibited EMT and the EGFR/ERK signaling pathway in ESCA cell lines.

### *FUT6* Suppressed the Proliferation, Migration, Invasion, and EMT of the ESCA Cell Lines via the EGFR/ERK Signaling Pathway

**Figure 4.**
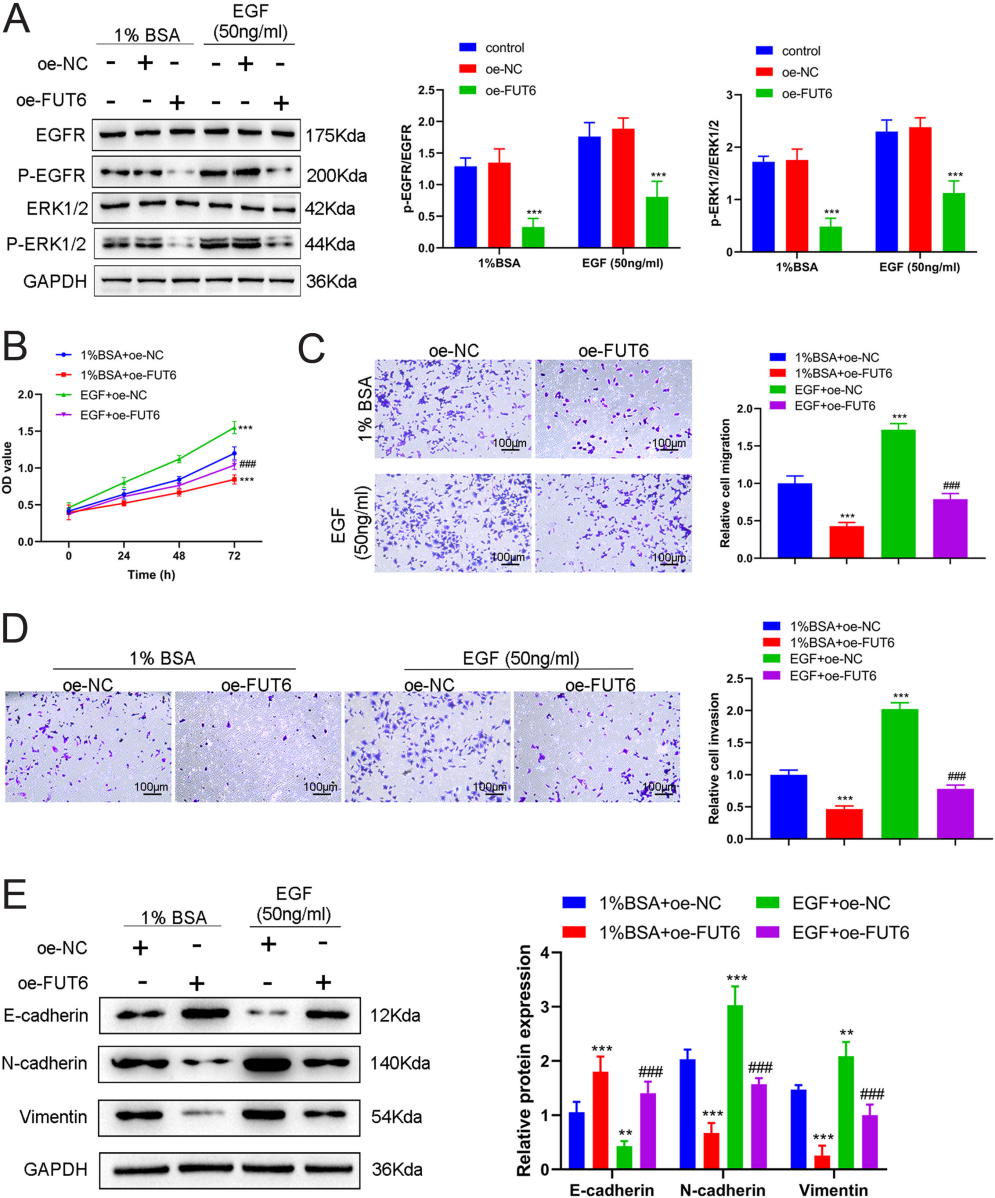
*FUT6* suppressed the proliferation, migration, invasion, and EMT of ESCA cells via the EGFR/ERK signaling pathway. (A) Western blotting of the protein expression levels of p-EGFR, EGFR, p-ERK1/2, and ERK1/2 in TE-1 cells transfected with oe-FUT6 in the presence of EGF (50 ng/mL) or 1% BSA, ***P < .001 vs. oe-NC group. (B) CCK-8 assay measurement of the proliferation of TE-1 cells transfected with oe-FUT6 in the presence of EGF (50 ng/mL) or 1% BSA. (C) Transwell assay measurement of the migration of TE-1 cells transfected with oe-FUT6 in the presence of EGF (50 ng/mL) or 1% BSA. (D) Transwell assay measurement of the invasion of TE-1 cells transfected with oe-FUT6 in the presence of EGF (50 ng/mL) or 1% BSA. (E) Western blotting of the protein expression levels of E-cadherin, N-cadherin, and Vimentin in TE-1 cells transfected with oe-FUT6 in the presence of EGF (50 ng/mL) or 1% BSA. Data: mean ± SEM. N = 3. **P < .01, ***P < .001 vs. 1% BSA + oe-NC group. ^###^ *P* < .001 vs. EGF + oe-NC group.

To gain a deeper comprehension of how *FUT6* controls EMT, migration, and invasion in ESCA cell lines, EGF was used as a stimulant to target the EGFR/ERK signaling pathway. Upon EGF stimulation, Western blotting results revealed significantly decreased protein levels of p-EGFR and p-ERK1/2 due to the notable overexpression of *FUT6*. Moreover, EGF treatment increased the protein levels of p-EGFR and p-ERK1/2 compared with the group treated with 1% BSA (Figure 4A). Further experiments on cell function demonstrated that EGF stimulation significantly enhanced not only cell proliferation (Figure 4B) but also migration (Figure 4C) and invasion (Figure 4D) of ESCA cells. Additionally, it increased the expression levels of E-cadherin and Vimentin proteins, while decreasing that of N-cadherin (Figure 4E). Notably, *FUT6* upregulation upon EGF stimulation significantly inhibited malignant cellular activities, including cell growth, movement, invasion, and EMT advancement (Figure 4B–E). The results suggest that *FUT6* effectively suppresses the malignant activity of ESCA cell lines by repressing the activation of the EGFR/ERK signaling pathway.

## Discussion

In the field of ESCA research, there have been many literature reports on the predictive value of various molecular markers for treatment response and prognosis of ESCA. The continuous development of genetic databases in recent years has opened up avenues of research where potential ESCA markers and molecular targets can be screened by identifying relevant differential genes.^11^ In this study, our data demonstrated that *FUT6* can impair the capabilities of proliferation, invasion, and migration of ESCA cell lines in vitro. Mechanistically, *FUT6* inhibits EMT of ESCA cell lines by suppressing the EGFR/ERK signaling pathway activated by EGF.

FUT6 belongs to the fucosyltransferase family, encoding α-1,3-fucosyltransferase, which catalyzes the transfer of fucosyltransferase from GDP-α-L-Fuc to FucT on the N-acetylglucosamine (GlcNAc) 3 position outside the sugar chain. It synthesizes the Lewis lineage antigens (Lea and Lex) and the sialylated Lewis lineage-associated antigens (sLea and sLex), which significantly affect tumor formation and progression.^12^ Li et al^9^ discovered a significantly lower *FUT6* expression level in breast cancer cell lines compared to the normal cell line. This decreased expression of *FUT6* was found to be involved in the movement, infiltration, and growth of human breast cancer cells. However, Liang et al^13^ discovered that *FUT6* could promote the growth, invasion, and angiogenesis of colorectal carcinomas via the miR-125a-3p/PI3K/Akt axis. Bai et al^14^ proposed that circSND1 activates *FUT6* expression through a mechanism that promotes the malignant behavior of cervical carcinomas. Furthermore, Guo et al^15^ characterized *FUT6* in human hepatocellular carcinoma and found that *FUT6* and its catalytic product, sLex, were strikingly increased in hepatocellular carcinoma tissues, and that *FUT6* upregulation in hepatocellular carcinomas enhanced S-phase cell populations and encouraged cell growth and colony-forming ability. The above illustrates the multifaceted nature of *FUT6* expression, which plays different roles in different tumors. This study revealed that ESCA cells exhibited a relatively low level of *FUT6* expression. Introducing the overexpression vector of *FUT6* through transfection hindered the proliferation, migration, and invasion of ESCA cells. Our findings provide an indication that *FUT6* acts as a tumor suppressor gene, exerting an anti-tumor effect on ESCA.

Epithelial-to-mesenchymal transition is crucial in spreading carcinomas to surrounding areas and infiltrating healthy tissues, serving as a pivotal factor in the progression of tumor cells toward malignancy.^16^ Epithelial-to-mesenchymal transition is a biological phenomenon in which epithelial cells are converted to have a mesenchymal phenotype through a specific program due to the loss of epithelial phenotypes such as loss of polarity and connectivity with surrounding cells and basement membranes, thereby acquiring a high capacity for migration, invasion, resistance to apoptosis, and degradation of the extracellular matrix.^17^ During the malignant evolution of ESCA, EMT allows tumor cells to infiltrate and metastasize and ESCA cells to escape apoptosis induced by certain factors.^18^ Research has confirmed that in ESCA cells, the occurrence of EMT primarily presents as a reduction in E-cadherin expression. This alteration allows the cells to acquire traits that facilitate invasion and metastasis. Consequently, the absence of E-cadherin was widely known as the most critical attributor of EMT.^19^ Additionally, EMT relies on the presence of certain crucial genes, including *Vimentin*, *Snail*, and *N-cadherin*, as significant indicators.^20^ Research has indicated that EMT in carcinomas encompasses various signaling pathways, such as the EGFR/ERK signaling pathway. The EGFR/ERK signaling pathway is known to play a crucial role in tumorigenesis due to its regulation of cell proliferation and differentiation.^21^ Studies have confirmed that the EGFR/ERK signaling pathway is overexpressed in ESCA tissues and plays a decisive role in ESCA pathophysiology. A study revealed that suppressing EGFR/ERK signaling pathway activation could promote apoptosis and enhance the radiosensitivity of ESCA cells.^22^ Zhao et al^23^ demonstrated that cetuximab can act as a potent radiosensitizer for ESCA due to its regulatory role in the EGFR/ERK signaling pathway. These results suggest that the malignant behavior of ESCA can be effectively repressed by treating the EGFR/ERK signaling pathway as a target to prevent its activation. In this study, we found that *FUT6* overexpression preeminently contributes to suppressing EMT and the EGFR/ERK signaling pathway, as evidenced by the decreased N-cadherin, Vimentin, p-EGFR, and p-ERK1/2 protein levels and the increased E-cadherin protein levels. Subsequently, EGF was introduced as an activator targeting the EGFR/ERK signaling pathway to further explore its regulation by *FUT6*. Currently, it is generally accepted that EGF is an important ligand capable of binding and activating EGFR.^24^ These findings indicated that *FUT6* overexpression significantly suppressed the malignant activities of cells, such as cell growth, movement, infiltration, and EMT advancement, following EGF stimulation. A previous study showed that *FUT6* overexpression in non-small-cell lung cancer cells affected the growth, movement, as well as infiltration of tumor. This increased the expression level of E-cadherin and decreased that of N-cadherin and Vimentin. Consequently, the levels of phosphorylation of EGFR, ERK, STAT, and c-Myc were ultimately reduced.^10^ It was confirmed that *FUT6* could regulate EMT and the EGFR/ERK signaling pathway during tumor progression. Accordingly, we propose the conclusion that *FUT6* suppresses the malignant function of ESCA cells by inhibiting the EGFR/ERK signaling pathway.

This study identified the ability of *FUT6* to regulate the malignant behavior of ESCA cells for the first time, providing a new understanding of the ESCA process and laying a certain foundation for the next in-depth study on the mechanism of the tumor suppressor effect of *FUT6* as well as the development of targeted therapies. However, this research had several limitations. First, this study is based on the cellular level, and animal experiments should be conducted to further validate the role of *FUT6* in ESCA xenograft mice. This study revealed that *FUT6* is relatively downregulated in ESCA cells, and whether *FUT6* expression profile is similarly in clinical tumorous samples of patients with ESCA needs to be confirmed by incorporating clinical trials.

In conclusion, this study demonstrated that *FUT6* can diminish the growth and invasion of ESCA cells under laboratory conditions. In terms of mechanism, *FUT6* hinders EMT in ESCA cells by repressing the EGFR/ERK signaling pathway triggered by EGF. Our findings provide a new understanding of ESCA development and lay a foundation for the next in-depth study on the mechanism of the tumor suppressor effect of *FUT6* as well as the development of targeted therapies.

## References

[1] Cao W Chen HD Yu YW Li N Chen WQ . Changing profiles of cancer burden worldwide and in China: a secondary analysis of the global cancer statistics 2020. Chin Med J (Engl). 2021;134(7):783 791. (10.1097/CM9.0000000000001474) 33734139 PMC8104205

[2] Zhu H Ma X Ye T , et al. Esophageal cancer in China: practice and research in the new era. Int J Cancer. 2023;152(9):1741 1751. (10.1002/ijc.34301) 36151861

[3] Bolger JC Donohoe CL Lowery M Reynolds JV . Advances in the curative management of oesophageal cancer. Br J Cancer. 2022;126(5):706 717. (10.1038/s41416-021-01485-9) 34675397 PMC8528946

[4] Zhou N Hofstetter WL . Prognostic and therapeutic molecular markers in the clinical management of esophageal cancer. Expert Rev Mol Diagn. 2020;20(4):401 411. (10.1080/14737159.2020.1731307) 32067548

[5] Lamouille S Xu J Derynck R . Molecular mechanisms of epithelial-mesenchymal transition. Nat Rev Mol Cell Biol. 2014;15(3):178 196. (10.1038/nrm3758) 24556840 PMC4240281

[6] Dongre A Weinberg RA . New insights into the mechanisms of epithelial-mesenchymal transition and implications for cancer. Nat Rev Mol Cell Biol. 2019;20(2):69 84. (10.1038/s41580-018-0080-4) 30459476

[7] Jayachandran J Srinivasan H Mani KP . Molecular mechanism involved in epithelial to mesenchymal transition. Arch Biochem Biophys. 2021;710:108984. (10.1016/j.abb.2021.108984) 34252392

[8] Scionti F Arbitrio M Caracciolo D , et al. Integration of DNA microarray with clinical and genomic data. Methods Mol Biol. 2022;2401:239 248. (10.1007/978-1-0716-1839-4_15) 34902132

[9] Li N Liu Y Miao Y Zhao L Zhou H Jia L . MicroRNA-106b targets FUT6 to promote cell migration, invasion, and proliferation in human breast cancer. IUBMB Life. 2016;68(9):764 775. (10.1002/iub.1541) 27519168

[10] Wang Q Liao C Tan Z , et al. FUT6 inhibits the proliferation, migration, invasion, and EGF-induced EMT of head and neck squamous cell carcinoma (HNSCC) by regulating EGFR/ERK/STAT signaling pathway. Cancer Gene Ther. 2023;30(1):182 191. (10.1038/s41417-022-00530-w) 36151332

[11] Song AY Mu L Dai XY Wang LJ Huang LQ . Analysis of significant genes and pathways in esophageal cancer based on gene expression omnibus database. Chin Med Sci J. 2023;38(1):20 28. (10.24920/004148) 36855320

[12] Puan KJ San Luis B Yusof N , et al. FUT6 deficiency compromises basophil function by selectively abrogating their sialyl-Lewis x expression. Commun Biol. 2021;4(1):832. (10.1038/s42003-021-02295-8) 34215830 PMC8253766

[13] Liang L Gao C Li Y , et al. miR-125a-3p/FUT5-FUT6 axis mediates colorectal cancer cell proliferation, migration, invasion and pathological angiogenesis via PI3K-Akt pathway. Cell Death Dis. 2017;8(8):e2968. (10.1038/cddis.2017.352) 28771224 PMC5596543

[14] Bai L Sun W Han Z Tang H . CircSND1 regulated by TNF-α promotes the migration and invasion of cervical cancer cells. Cancer Manag Res. 2021;13:259 275. (10.2147/CMAR.S289032) 33469369 PMC7811455

[15] Guo Q Guo B Wang Y , et al. Functional analysis of α1,3/4-fucosyltransferase VI in human hepatocellular carcinoma cells. Biochem Biophys Res Commun. 2012;417(1):311 317. (10.1016/j.bbrc.2011.11.106) 22155250

[16] Pastushenko I Blanpain C . EMT Transition states during tumor progression and metastasis. Trends Cell Biol. 2019;29(3):212 226. (10.1016/j.tcb.2018.12.001) 30594349

[17] Jinesh GG Brohl AS . Classical epithelial-mesenchymal transition (EMT) and alternative cell death process-driven blebbishield metastatic-witch (BMW) pathways to cancer metastasis. Signal Transduct Target Ther. 2022;7(1):296. (10.1038/s41392-022-01132-6) 35999218 PMC9399134

[18] Li M Li X Chen S , et al. IPO5 mediates EMT and promotes esophageal cancer development through the RAS-ERK pathway. Oxid Med Cell Longev. 2022;2022:6570879. (10.1155/2022/6570879) 36120598 PMC9481360

[19] Zhao H Hu H Chen B , et al. Overview on the role of E-cadherin in gastric cancer: dysregulation and clinical implications. Front Mol Biosci. 2021;8:689139. (10.3389/fmolb.2021.689139) 34422902 PMC8371966

[20] Kong P Xu E Bi Y , et al. Novel ESCC-related gene ZNF750 as potential Prognostic biomarker and inhibits Epithelial-Mesenchymal Transition through directly depressing SNAI1 promoter in ESCC. Theranostics. 2020;10(4):1798 1813. (10.7150/thno.38210) 32042337 PMC6993233

[21] Levantini E Maroni G Del Re M Tenen DG . EGFR signaling pathway as therapeutic target in human cancers. Semin Cancer Biol. 2022;85:253 275. (10.1016/j.semcancer.2022.04.002) 35427766

[22] Yang QS Jiang LP He CY Tong YN Liu YY . Up-regulation of MicroRNA-133a inhibits the MEK/ERK signaling pathway to promote cell apoptosis and enhance radio-sensitivity by targeting EGFR in esophageal cancer in vivo and in vitro. J Cell Biochem. 2017;118(9):2625 2634. (10.1002/jcb.25829) 27933650

[23] Zhao G Feng L Ye T , et al. Cetuximab enhances radiosensitivity of esophageal squamous cell carcinoma cells by G2/M cycle arrest and DNA repair delay through inhibiting p-EGFR and p-ERK. Thorac Cancer. 2023;14(22):2127 2138. (10.1111/1759-7714.14995) 37337933 PMC10396788

[24] Sabbah DA Hajjo R Sweidan K . Review on epidermal growth factor receptor (EGFR) structure, signaling pathways, interactions, and recent updates of EGFR inhibitors. Curr Top Med Chem. 2020;20(10):815 834. (10.2174/1568026620666200303123102) 32124699

